# Positive response of a hemodialysis patient with pure red cell aplasia on recombinant human erythropoietin therapy to cyclosporine and Roxadustat

**DOI:** 10.1007/s13730-024-00865-3

**Published:** 2024-03-25

**Authors:** Xuejuan Ma, Pearl Pai, Wenjuan Zhu, Xiaowei Chen, Liwen Cui

**Affiliations:** 1 Department of Nephrology, The University of Hong Kong-Shenzhen Hospital, Shenzhen, China; 2 The First Clinical Medical College of Jinan University, Guangzhou, Guangdong China; 3 Department of Medicine, University of Hong Kong Li Ka Shing Faculty of Medicine, Hong Kong, China; 4 Department of Medicine, The University of Hong Kong-Shenzhen Hospital, Shenzhen, 518053 Guangdong People’s Republic of China

**Keywords:** Dialysis, Erythropoetin, Pure red cell aplasia, Cyclosporine, Roxadustat

## Abstract

Recombinant human erythropoietin (rHuEPO) is commonly used to treat anemia associated with chronic kidney disease (CKD). EPO-induced Pure Red Cell Aplasia (PRCA) is a rare condition of profound anemia with EPO treatment. Upon finding the development of EPO-induced PRCA, the treatment requires immediate withdrawal of EPO therapy and initiate new treatments with immunosuppression or renal transplantation. Anti-EPO antibody assay is not always positive in EPO-induced PRCA. Here, we report a case on the sudden development of PRCA in a hemodialysis patient receiving EPO and how we treated the condition successfully with cyclosporine and subsequently maintained the hemoglobin with Roxadustat, a hypoxia-inducible factor prolyl hydroxylase inhibitor (HIF-PHI). Even though the anti-EPO antibody was negative by Enzyme Linked Immunosorbent Assay (ELISA) in our case, the clinical course, the markedly reduced reticulocyte count < 10,000/μL, the bone marrow (BM) biopsy revealing reduced erythroblasts, and its subsequent response to cyclosporine, were similar to EPO-induced PRCA. The clinical picture of EPO-induced PRCA, the limitation of the EPO-neutralizing antibody (Ab) assay, and treatment strategies were discussed.

## Introduction

Recombinant human erythropoietin (rHuEPO) effectively treats renal anemia in chronic kidney disease (CKD) patients. The treatment can lead to a number of complications. EPO-induced Pure Red Cell Aplasia (PRCA) is one of the very rare complications, with an incidence of 0.02–0.03 per 10,000 patient years [1]. Often, the anemia occurs suddenly due to the development of EPO-neutralizing antibodies (Abs) eliminating the efficacy of both exogenous and endogenous EPO. Hypoxia-inducible factor (HIF), a hypoxia-regulated transcription factor, undergoes rapid degradation under normoxic conditions. HIF-prolyl hydroxylase inhibitor (PHI) stabilizes HIF and stimulates the expression of HIF target genes, including the EPO gene [2]. Roxadustat, an oral HIF-PHI, improves iron bioavailability, stimulates endogenous EPO, and corrects anemia of renal disease. We hereby present a case of a hemodialysis (HD) patient who clinically developed PRCA after 10 months of stable EPO therapy. A switch from rHuEPO to Roxadustat treatment was not effective early on, but the patient responded after 2 months of cyclosporine therapy.

## Case report

A 58-year-old male patient was diagnosed with CKD secondary to diabetes mellitus. In July 2021, he was commenced on rHuEPO (Jimaixin, epoetin-α, KexingBio, Shandong, China) subcutaneously 5000 IU twice a week. At the time of his EPO therapy, his hemoglobin (Hb) was 99 g/L. He commenced HD in November 2021. For a start, he was treated with intravenous (iv) rHuEPO (6000 IU) (Yipuding, epoetin-α, NCPC GenetechBio, Hebei, China) once a week and his Hb increased to 116 g/L over 3 months. In May 2022, the patient complained of sudden fatigue. His local HD unit increased his rHuEPO to iv 6000 IU thrice a week without checking his full blood count. On 21 May 2022, his Hb was reported as 75 g/L, and his rHuEPO was adjusted to subcutaneous 6000 IU thrice a week. But his fatigue persisted, leading to his admission to our hospital on 18 June 2022, with a very low Hb level of 48 g/L. He was given a total of 6.5 units of red blood cell (RBC) transfusion. The rHuEPO was stopped and his prescription was changed to Roxadustat 100 mg thrice a week. Shortly afterward, he was found to have a pneumonia. He underwent bronchoalveolar lavage (BAL) and responded to a 10-day course of Piperacillin-Tazobactam and Cefuroxime. On 27 June 2022, the dosage of Roxadustat was increased to 150 mg, thrice weekly. The patient's Hb level was stable at 67–69 g/L and he was discharged on Roxadustat after a week. The EPO level taken on 28 June was subsequently reported as 22.3 mIU/ml (normal range 4.3–29 mIU/ml).

Only after 18 days, on 20 July 2022, the patient was readmitted. His red blood cells were 1.42 × 10^12/L, Hb 38 g/L, hematocrit 10.7%, reticulocytes 2900/μL, white blood count 8.35 × 10^9/L, platelet count 150 × 10^9/L. Due to the unexplained and persistent severe anemia, a BM biopsy was undertaken on 22 July 2022 which indicated a proliferative active bone marrow but reduced erythroblasts of 7%. A diagnosis of EPO-induced PRCA was suspected. Some of the likely causes of PRCA such as lymphoproliferative disorder, parvovirus B19 infection, thymoma and systemic autoimmune disease were ruled out. There were adequate iron store, vitamin B12 level, and folic acid. C-reactive protein (CRP) was 2.06 ng/ml. The chest and abdominal CT scan and gastroscopy did not show any space-occupying lesion or bleeding. Based on the clinical course and laboratory findings, a case of an EPO-induced PRCA was strongly suspected and an anti-EPO Ab test was ordered. However, the test via the Enzyme Linked Immunosorbent Assay (ELISA) method returned negative. Roxadustat was discontinued on 20 July 2022. On 4 August 2022, the patient was started on cyclosporine to try and aim for a trough level of 100–150 ng/mL. The patient received 12 units of RBC transfusion before he was discharged on 9 August 2022.

**Fig. 1:**
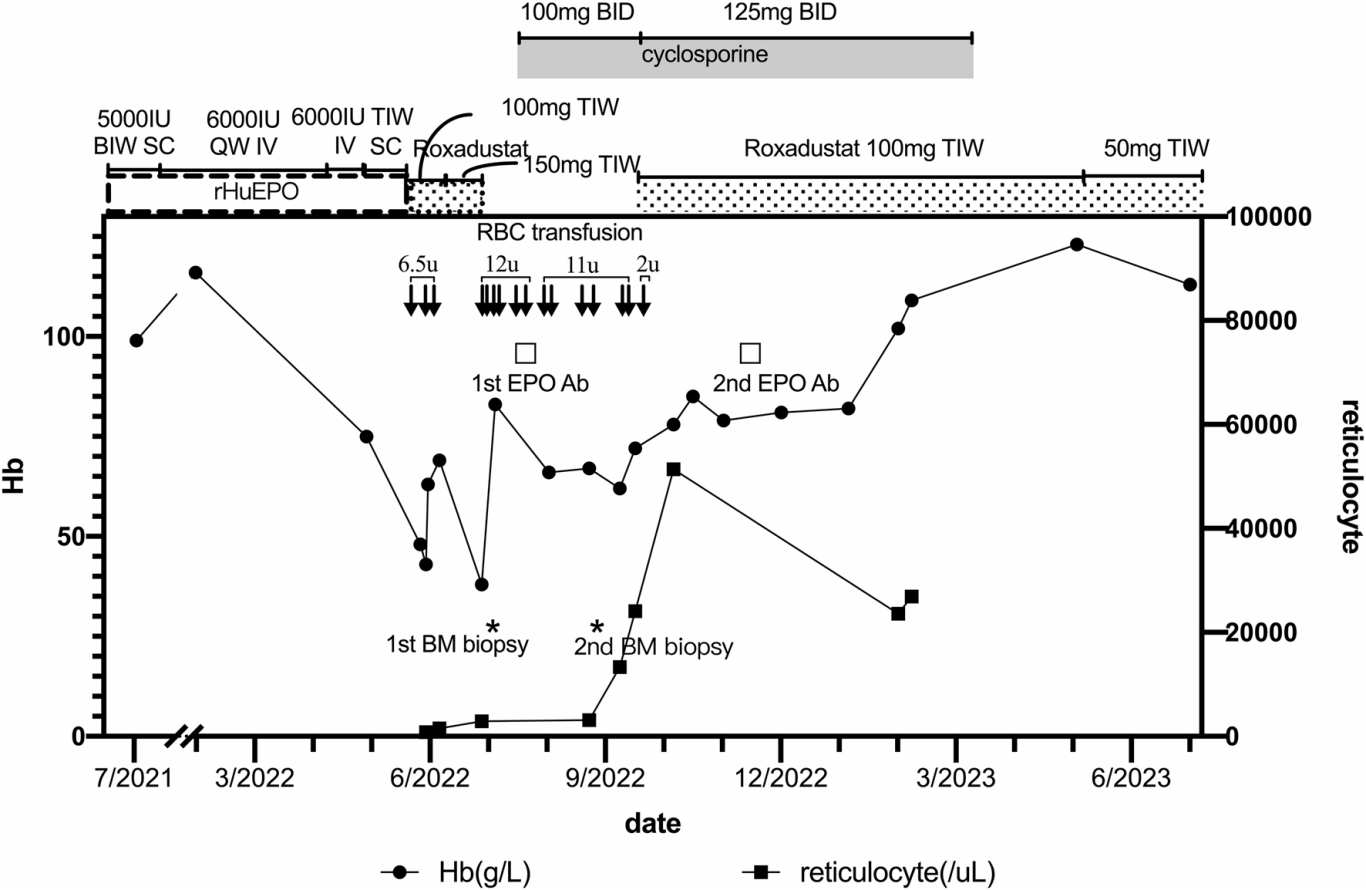
Clinical course of the patient showing a sudden decrease of hemoglobin (Hb) level in May 2022. The EPO therapy was changed to Roxadustat in June 2022 with no response. Roxadustat was discontinued in July 2022 and cyclosporine was initiated in August 2022 after a clinical diagnosis of pure red cell aplasia on EPO was made. Bone marrow biopsy was performed in July and September 2022, respectively (*). The patient received red blood cell transfusion 16 times totaling 31.5u. EPO antibody was tested in August and December 2022, respectively (囗). As reticulocyte count increased, Roxadustat was recommenced to treat anemia of chronic renal disease in October 2022. *RBC* red blood cell, *Hb* hemoglobin, *BM* bone marrow, *Ab* antibody, *IU* international unit, *IV* intravenous, *SC* subcutaneous injection, *QW* once a week, *BIW* twice a week, *TIW* thrice a week, *BID* twice a day, *u* unit

Between 9 August until 7 October 2022, the patient had required 11 units of RBC transfusion to maintain a Hb of 70–80 g/L. A second BM biopsy undertaken on 20 September showed a further decrease of erythroblasts to 5.2%. The reticulocyte count started to rise toward end of September and early October 2022. Roxadustat was reintroduced on 9 October 2022 at a dosage of 100 mg thrice weekly. His last RBC transfusion (2 units) was given on 14 October 2022. Thereafter, the patient had maintained a stable Hb levels between 73 and 89 g/L and in no need of further RBC transfusion. Subsequent analysis of EPO levels prior to the RBC transfusion on 14 October showed a value of 161 mIU/ml (normal range 4.3–29 mIU/ml). A second anti-EPO-Ab ELISA test taken on 9 December 2022 was negative. The cyclosporine was stopped in March 2023 and the follow-up Hb was 113 g/L in July 2023. The treatment course is summarized in Fig. 1.

## Discussion

The first case report of EPO-induced PRCA was published in the late 1990s. Reports on the condition reached its peak in 2001 and 2002 [3] and continued to appear intermittently thereafter. During treatment with EPO, a sudden decrease in Hb levels, accompanied by markedly reduced reticulocyte count, suggests the possibility of EPO-induced PRCA [4]. The following criteria need to be met for diagnosis of EPO-induced PRCA: (i) the common causes of PRCA have been excluded; (ii) less than 5% of erythroblasts in the bone marrow with otherwise normal cellularity; and (iii) positive antibodies binding EPO detected by available tests [4].

In the first hospital admission of our patient, we considered his severe anemia a result of severe infection and hyporesponsiveness to rHuEPO. As past case report had shown the efficacy of Roxadustat in treating EPO-hyporesponsive anemia [5], we changed his rHuEPO to Roxadustat and his infection was brought under control quickly. His Hb was stable for quite a few days without RBC transfusion and he was discharged.

But during his second hospital admission, his anemia was more severe. By that time, his chest infection was under control and his CRP had normalized. We excluded other causes such as iron deficiency, on-going or latent infection, tumor, bleeding and hemolysis for his severe anemia. The more common causes of PRCA such as lymphocyte and plasma cell disorders, autoimmune disorders, viral infection, thymoma or other suspected medication were ruled out, too. Hence, we felt the sudden loss of efficacy to rHuEPO and a reduction of reticulocytes < 10,000/μL were highly suggestive of EPO-induced PRCA [6]. Meanwhile, the second BM biopsy showed erythroblasts of 5.2%, dropping further from 7% obtained in the first biopsy. Although the percentage of erythroblasts was just over the 5% cut off for PRCA, as the disease progressed, we speculated the erythroblasts would fall below 5%.

Our patient’s EPO-binding Ab test was negative to our surprise. Our literature search found two other case reports of PRCA induced by EPO therapy without demonstrable EPO antibodies [7, 8]. In our case, we speculated a number of plausible reasons for the absence of proven EPO Abs. The recommended assays for identifying EPO Abs included radioimmunoprecipitation (RIP), ELISA, surface plasmon resonance (BIAcore), and bioassays [9]. Each of these tests came with its own advantages and disadvantages in terms of sensitivity, specificity and accessibility [9]. In China, the available tests are limited and we have only been able to use an ELISA method which is relatively inexpensive and easy to use. EPO is combined with a porous plate and a secondary Ab is used to detect the binding of Abs in patient’s serum [6]. Depending on the washing conditions, Abs may bind non-specifically to the plate, whereas low-affinity Abs may be washed away resulting in low specificity and sensitivity of the conventional ELISA assay [6]. The sensitivity of EPO-Ab test may also be affected by timing of the sampling in relation to cessation of EPO therapy or commencement of Roxadustat. In a case series of 13 patients with EPO-induced PRCA, after discontinuation of EPO therapy, the levels of EPO Abs in the patients declined gradually [10]. It has also been speculated that endogenous EPO upregulated by Roxadustat helps neutralize anti-EPO Abs without boosting the formation of EPO Abs [11]. In our case, the first blood sample for EPO-Abs was taken after the patient’s EPO therapy had been stopped and Roxadustat commenced for a month. We speculated that our negative Ab results might be due to the low sensitivity of the ELISA method and/or diminishing level of EPO-Abs. While we suspected that the cause of PRCA in our case was the result of EPO Abs that was not yet made demonstrable, there remained a slight possibility that the PRCA had been due to another cause responding to cyclosporine [12].

Regarding therapy, once EPO-induced PRCA is diagnosed, merely stopping the EPO is often not enough for the recovery of red blood cell production. Such recovery has been reported in patients after treatment with immunosuppressive therapy or renal transplant [13]. Both glucocorticoid and cyclosporine have been shown to be effective in treating renal patients with EPO-induced PRCA [12]. In this case, we have managed to use cyclosporine alone and observed a gradual recovery of reticulocyte counts. We have chosen to avoid steroid to reduce hyperglycemia and the regimen has worked.

In recent years, Roxadustat, an oral HIF-PHI, has demonstrated efficacy and safety in the treatment of kidney disease patients with renal anemia whether on dialysis or not [14, 15]. In our literature search, we found a case of dialysis patient whose EPO-induced PRCA resolved after 3 months of treatment with steroids and cyclosporine, followed by Roxadustat [16]. In another case, the anemia responded to Roxadustat after 5 months of cyclosporine therapy [17].

It is likely that the erythropoietic response to Roxadustat after cyclosporine is related to the mechanistic action of Roxadustat. Roxadustat increases endogenous EPO levels by stabilizing HIF-α that trigger EPO genes through HIF-PHI. However, in patients with EPO-induced PRCA and high Ab levels, we speculate that Roxadustat on its own may be ineffective early on due to the high level of anti-EPO Abs interacting with both endogenous and exogenous EPO [16]. In these cases, only after adequate immunosuppressive treatment and reduction of Ab titers can Roxadustat promote haematopoiesis and alleviate the anemia. It is not clear whether the patient may be safely re-challenged with EPO or he may suffer a relapse [18]. The option of using a HIF-PHI is a challenge and trial. In our case, we chose to use Roxadustat after 2 months of cyclosporine. We have used cyclosporine 250 mg/day in two divided doses to aim to achieve a trough level of 100–150 ng/mL. Steroid has been avoided to reduce hyperglycemia. As the reticulocyte counts rose over the next couple of months, Roxadustat was introduced to stimulate and maintain the erythropoiesis of the patient.

In conclusion, we could say that we have encountered and treated successfully a HD patient who developed PRCA whilst on EPO therapy with negative Ab under an ELISA test. The clinical course, markedly reduced reticulocyte count < 10,000/μL, the BM biopsy revealing reduced erythroblasts, and its subsequent response to cyclosporine, were in keeping with EPO-induced PRCA. Anti-EPO Ab assay is not always positive in EPO-induced PRCA and to which the clinicians should be alerted. With or without demonstrable EPO Abs, our case showed that treatment with cyclosporine and a switch to Roxadustat might be proven and effective in treating this type of severe anemia in patients treated with EPO previously.
